# Hypercalcemia Unveiled by Hidden Vitamin A Overload in an Elderly Woman

**DOI:** 10.7759/cureus.76816

**Published:** 2025-01-02

**Authors:** Marwah Alchalabi, Dinara Salimova, Mohamed A Ebrahim, Ricardo Loor-Torres, Faisal Qureshi

**Affiliations:** 1 Internal Medicine, Ascension Saint Joseph Hospital, Chicago, USA; 2 Endocrinology, Diabetes and Metabolism, Ascension Saint Joseph Hospital, Chicago, USA

**Keywords:** abnormal bone turnover, hypercalcemia, secondary hypercalcemia, vitamin a supplements, vitamin a toxicity

## Abstract

Vitamin A is a commonly available supplement found naturally in many foods. While it is essential for numerous physiological processes, caution is required when supplementing at high doses, as excessive intake can result in vitamin A toxicity, which may present with hypercalcemia. We report a case of a patient who developed elevated calcium levels associated with excessive vitamin A consumption. This case highlights the importance of considering inappropriate vitamin A supplementation as a potential cause of hypercalcemia.

## Introduction

Shaw and Niccol reported the first case of hypercalcemia induced by hypervitaminosis A in 1953 [1]. Vitamin A is commonly used to treat many conditions, including psoriasis, acne vulgaris, and measles [2,3]. It is found ubiquitously in both animal- and plant-based sources. The two primary forms of vitamin A are preformed vitamin A, which includes retinol, retinal, retinoic acid, and retinyl esters, and provitamin A, primarily represented by carotenoids [4]. In addition, many over-the-counter (OTC) supplements, which have grown in popularity in recent years, also contain vitamin A. This is particularly relevant, as nearly 50% of the U.S. population reports using OTC supplements, with about one-third taking multivitamins that include vitamin A [5].

Hypercalcemia is a prevalent condition, affecting up to 1 in 1,000 individuals in the general population. It is most common causes are primary hyperparathyroidism or malignancy, although various other etiologies exist, including excessive vitamin A intake. In developed countries, the classic symptomatic presentation of hypercalcemia is relatively uncommon, with the condition more often being identified incidentally through biochemical testing in asymptomatic individuals [6].

Vitamin A is crucial in several essential biological functions, including growth, vision, gene expression, skin health, wound healing, and immune system support. Both deficiency and excess of vitamin A can lead to significant health issues. Deficiency is more common, particularly among pediatric and pregnant populations, and it can result in serious complications. Conversely, while rare, vitamin A hypervitaminosis can cause adverse effects such as birth defects, hair loss, and hypercalcemia [7,8]. This article describes a case of hypercalcemia caused by excessive vitamin A supplementation.

## Case presentation

A 69-year-old female was incidentally noted to have serum calcium levels of 11.2 mg/dL (8.8 - 10.2 mg/dL) by her primary care physician and was referred to our endocrinology clinic for further work-up. She had a significant medical history of metastatic estrogen receptor-positive, progesterone receptor-positive, and human epidermal growth factor receptor 2 (Her2)-negative stage IV invasive ductal breast cancer, which was diagnosed approximately eight years before this visit. At that time, imaging studies had shown osteoblastic metastatic lesions of the pelvic bone and liver. She has received multiple cycles of chemotherapy and has been receiving oral capecitabine (500 mg twice daily) over the last 2 years. In addition, she also has a history of uterine fibroids, hepatosteatosis, arterial hypertension, dyslipidemia, and left knee osteoarthritis. Her medications included amlodipine 5 mg daily, atorvastatin 40 mg daily, cyanocobalamin 100 mcg daily, gabapentin 100 mg three times daily, and docusate** **100 mg needed for constipation, as well as denosumab injections every three months for treatment of bone metastases. Her family history was unremarkable for hypercalcemia or nephrolithiasis.

During the initial visit, the patient complained about chronic fatigue, nausea, and constipation that has not responded to bulk-producing laxatives such as docusate. She denied abdominal or flank pain, muscle spasms, headache, polyuria, or changes in mood or mental status. Notably, upon interrogation, the patient reported that she started to take 25,000 units of vitamin A supplementation four months before the initial consult. Otherwise, she denied taking additional calcium supplements, calcium carbonate, or vitamin D. At the visit, vital signs were unremarkable and showed a temperature of 97.8 F, a heart rate of 70 beats per minute, a blood pressure of 134/75 mm Hg, a respiratory rate of 14 breaths per minute, and normal oxygen saturation on room air. The physical examination was unremarkable.

Repeated blood work showed a calcium level of 11.2 mg/dL and a parathyroid hormone level of 19.0 pg/mL (15.0 - 72.0 pg/mL). The parathyroid hormone-related peptide was less than 0.4 pmol/L, and 24-hour urine calcium was slightly elevated. Other tests, such as serum protein electrophoresis, urine protein electrophoresis, 25-hydroxyvitamin D, 1,25 - dihydroxyvitamin D, and thyroid-stimulating hormone, were within normal limits. The patient had normal renal function. Of note, despite the patient being presented with osteoblastic bone metastases, they have been stable, and she had never experienced hypercalcemia in the past. For instance, one month before she visited our endocrinology clinic, her total serum calcium was 10.0 mg/dL, and her parathyroid hormone level was 29.4 pg/mL. Also, six months before her presentation, total serum calcium was 9.8 mg/dL. Table 1 provides a summary of her laboratory workup.

**Table 1:** A summary of laboratory workup

	Values	Units	Reference values
Total calcium, blood (at the visit)	11.2	mg/dL	8.8 - 10.2
Corrected calcium, blood (at the visit)	11.2	mg/dL	8.8 - 10.2
Parathyroid hormone (PTH)	19.0	pg/mL	15.0 - 72.0
Thyroid-stimulating hormone (TSH)	1.38	IU/mL	0.270 - 4.200
Serum creatinine	0.64	mg/dL	0.7 - 1.3
Glomerular filtration rate (GFR)	> 60	mL/min/1.73m2	> 60
25-hydroxyvitamin D	45	ng/mL	30 - 100
Total calcium, blood (1 month prior to visit)	10.0	mg/dL	8.8 - 10.2
Corrected calcium, blood (1 month prior to visit)	10.0	mg/dL	8.8 - 10.2
Parathyroid hormone - related peptide (PTHrP)	< 0.4	pmol/L	< or = 4.2
Total protein	7.8	g/dL	6.4 - 8.9
Albumin	4.5	g/dL	3.5 - 5.7
Urine calcium	302	mg/24 hours	100-300
Aspartate aminotransferase (AST)	27	IU/L	13 - 39
Alanine aminotransferase (ALT)	23	IU/L	7.0 - 52.0
Serum phosphorus	3.7	mg/dL	2.5 - 4.5
Alkaline phosphatase	77	IU/L	35-104
Total bilirubin	0.6	mg/dL	0.3-1.0

**Figure 1:**
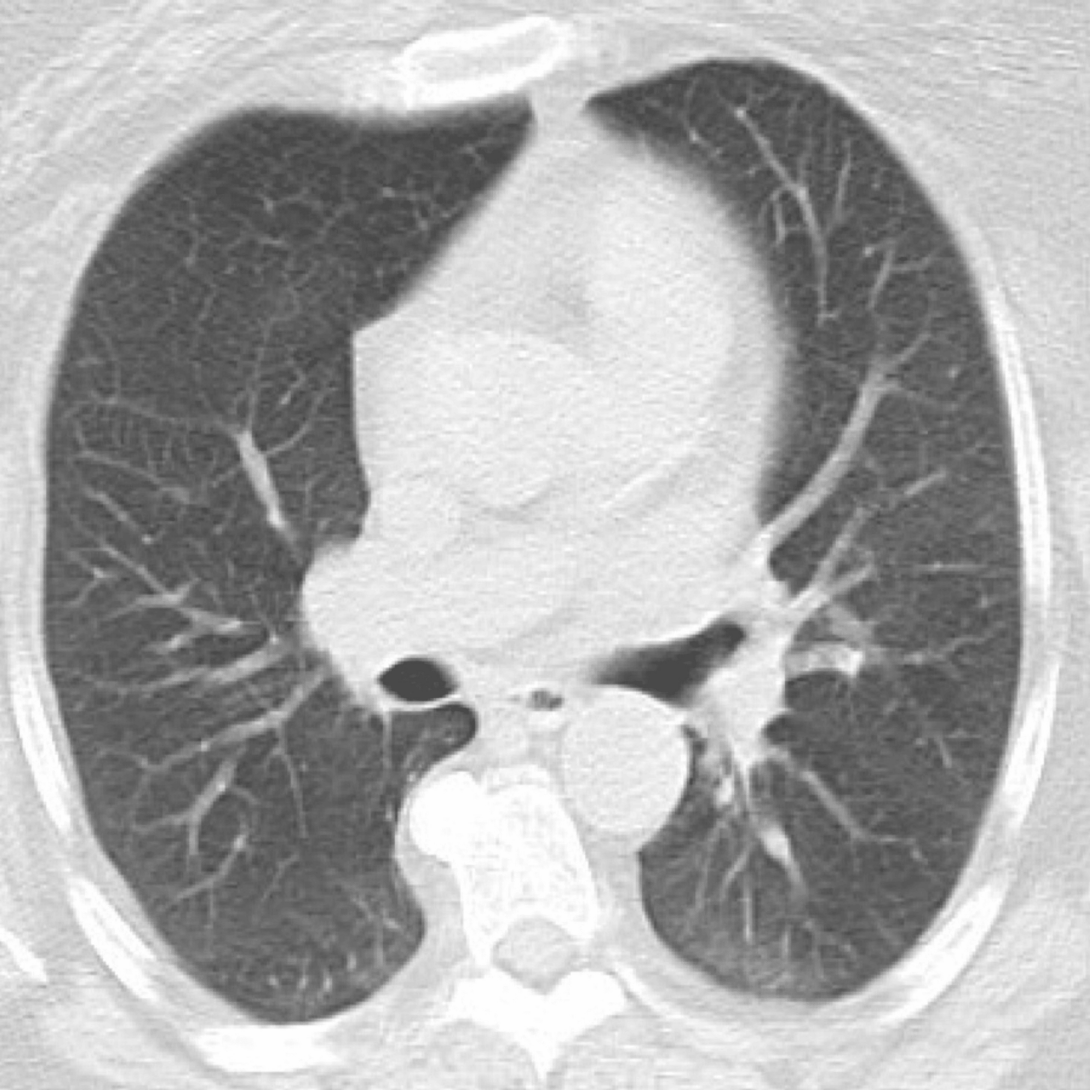
CT scan of the chest without contrast

A dual X-ray absorptiometry (DXA) scan performed one month prior to presentation showed no evidence of osteopenia or osteoporosis. While the patient did not have a chest X-ray in the months leading up to her presentation with hypercalcemia, a computed tomography (CT) scan of the chest conducted within a month of her initial visit to the endocrinologist revealed no lung metastases, stable bone sclerotic lesions, and was negative for sarcoidosis or lymphadenopathy, and the bone scan demonstrated stable osteoblastic metastatic disease. The picture below provides an image of the CT scan of the chest without contrast (Figure 1).

Based on the available information, the patient was diagnosed with hypercalcemia attributed to vitamin A supplementation and was advised to discontinue the supplement. One month after stopping vitamin A, her calcium level decreased to 9.5 mg/dL and remained stable during follow-up visits with her primary care physician.

## Discussion

Primary hyperparathyroidism and malignancy are the most common causes of hypercalcemia. Other less common etiologies include familial hypocalciuric hypercalcemia and granulomatous disorders. However, hypervitaminosis A, although rare, can represent a diagnostic challenge as it can be overlooked as a potential cause of hypercalcemia [6]. Given its variable clinical presentation and the growing use of over-the-counter vitamin supplements, including vitamin A, it is important to highlight this case. 

Our patient presented with chronic fatigue, nausea, and new-onset constipation unresponsive to bulk-forming laxatives. An initial evaluation, including a thorough medical history and physical examination, was unremarkable. Laboratory findings showed elevated serum calcium levels. Further work-up revealed plasma intact parathyroid hormone (PTH) levels within the lower normal range, ruling out primary hyperparathyroidism. Additional investigations, such as parathyroid hormone-related protein (PTHrP), 25-hydroxyvitamin D, urine protein electrophoresis, serum protein electrophoresis, and a chest X-ray, were also unremarkable. The patient presented with stable osteoblastic bone lesions with no apparent changes, and her calcium levels had consistently been within normal ranges during prior evaluations. Importantly, she disclosed initiating a daily intake of 25,000 IU of vitamin A supplementation several months earlier, encouraged by literature suggesting potential survival benefits in breast cancer patients.

While the exact mechanism underlying vitamin A-induced hypercalcemia has not been fully elucidated, it is believed to involve increased osteoclastic activity, reduced osteoid formation, suppression of osteoblastic activity, and impaired vitamin D receptor function [9]. A review of the literature identifies five groups of individuals at risk for developing hypercalcemia due to vitamin A toxicity: (I) patients with acute promyelocytic leukemia receiving all-trans retinoic acid (ATRA); (II) patients with chronic renal disease, even those not on hemodialysis, who are taking over-the-counter vitamin A; (III) patients receiving enteral tube feeding; (IV) elderly individuals taking excessive over-the-counter vitamin A supplements; and (V) patients with burns who are given steroids along with vitamin A to promote healing [5, 9, 10-12]. 

Vitamin A metabolism primarily occurs in the small intestine, where bile acids aid in absorption in the duodenum. Once absorbed, the esterified form of vitamin A travels to the liver cells. In the liver, retinol is de-esterified and then binds to retinol-binding protein, which facilitates its release to tissues as needed. Preformed vitamin A is absorbed approximately 70-90% efficiently, while carotenoids are absorbed at a significantly lower rate, ranging from 9% to 22%. Therefore, compared to preformed (artificial) vitamin A, carotenoids derived from plant sources are less likely to cause toxicity [4,10,13]. The recommended daily allowance (RDA) of vitamin A for adults is 700 micrograms for women and 900 micrograms for men. Remarkably, one microgram of vitamin A is equivalent to 1 RDA of retinoids. Ingesting more than 200 mg of the active form in a single dose can cause acute hypercalcemia, while chronic hypercalcemia occurs when the intake surpasses 10 times the RDA over an extended period. Since vitamin A is stored in the liver and excreted by the kidneys, individuals with renal insufficiency, hepatic dysfunction, or excessive alcohol consumption may experience reduced tolerance to the vitamin [4,14].

Toxic effects of hypervitaminosis A involve several organs and mainly include the bone, liver, and skin. According to the severity, it causes nausea, vomiting, cheilosis, and alopecia in mild to moderate cases and pseudotumor cerebri, which can manifest by altered cognition, muscle weakness, headaches, and visual disturbances due to elevated intracranial pressure in severe cases [4,13].

Treating vitamin A toxicity primarily involves discontinuing vitamin A intake and providing supportive care, such as ensuring adequate fluid intake. In cases of severe hypercalcemia, inpatient treatment may include the administration of calcitonin, corticosteroids, and activated charcoal to prevent further absorption of vitamin A. For mild to moderate toxicity, calcium levels typically normalize after cessation of vitamin A. Patient education and dietary monitoring are essential components of care that reduce the risk of future toxicity [15].

## Conclusions

Chronic excessive consumption of vitamin A can lead to hypercalcemia due to various mechanisms, including increased bone turnover. A comprehensive evaluation is crucial to differentiate vitamin A toxicity from other potential causes of hypercalcemia. Extra caution is warranted when using over-the-counter vitamin A supplements, particularly among elderly individuals who may be at an elevated risk for adverse effects.
